# Geographic variation and thermal plasticity shape salamander metabolic rates under current and future climates

**DOI:** 10.1002/ece3.8433

**Published:** 2022-01-15

**Authors:** David Muñoz, David Miller, Rudolf Schilder, Evan H. Campbell Grant

**Affiliations:** ^1^ Department of Ecosystem Science and Management The Pennsylvania State University University Park Pennsylvania USA; ^2^ Department of Entomology Department of Biology The Pennsylvania State University University Park Pennsylvania USA; ^3^ US Geological Survey Patuxent Wildlife Research Center SO Conte Anadromous Fish Research Lab Turners Falls Massachusetts USA

**Keywords:** climate change, intraspecific variation, metabolic rate, plasticity

## Abstract

Predicted changes in global temperature are expected to increase extinction risk for ectotherms, primarily through increased metabolic rates. Higher metabolic rates generate increased maintenance energy costs which are a major component of energy budgets. Organisms often employ plastic or evolutionary (e.g., local adaptation) mechanisms to optimize metabolic rate with respect to their environment. We examined relationships between temperature and standard metabolic rate across four populations of a widespread amphibian species to determine if populations vary in metabolic response and if their metabolic rates are plastic to seasonal thermal cues. Populations from warmer climates lowered metabolic rates when acclimating to summer temperatures as compared to spring temperatures. This may act as an energy saving mechanism during the warmest time of the year. No such plasticity was evident in populations from cooler climates. Both juvenile and adult salamanders exhibited metabolic plasticity. Although some populations responded to historic climate thermal cues, no populations showed plastic metabolic rate responses to future climate temperatures, indicating there are constraints on plastic responses. We postulate that impacts of warming will likely impact the energy budgets of salamanders, potentially affecting key demographic rates, such as individual growth and investment in reproduction.

## Graphical abstract

We find geographic variation in thermal plasticity of metabolic rate in a widespread salamander. This variation in metabolic is an energy‐saving mechanism under future climates, but is currently constrained and is unlikely to support population persistence under future climates.

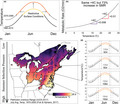

## 1 INTRODUCTION

Tools to predict organismal responses to climate change increasingly incorporate ecological and physiological traits such as energy balance and life history (Buckley, 2008; Kearney & Porter, 2009; Urban et al., 2016). However, most models ignore variation in such traits among populations or life stages (Cotto et al., 2017; Sinclair et al., 2016; Urban et al., 2016). This is despite evidence that intraspecific variation in traits is ecologically relevant (Des Roches et al., 2018) and that accounting for within‐species variation is critical for predicting outcomes under future climates (Leites et al., 2012; McCain et al., 2016). Variation among populations or life stages can occur because of local adaptation, ontogenetic ecological differences, or plasticity. These types of variation may constrain population responses (Valladares et al., 2014), increase the breadth of necessary conditions for population persistence (Riddell et al., 2018a), or maintain ecological performance across a wide range of environmental conditions, respectively (Huey et al., 2012). Given that populations experience different selection pressures and have different evolutionary histories, we should expect variation to be the norm (Peterson et al., 2019; Sexton et al., 2009) and should use patterns of variability to critically inform how species may respond to a changing climate (Franklin, 2010; Moran et al., 2015).

**FIGURE 1:**
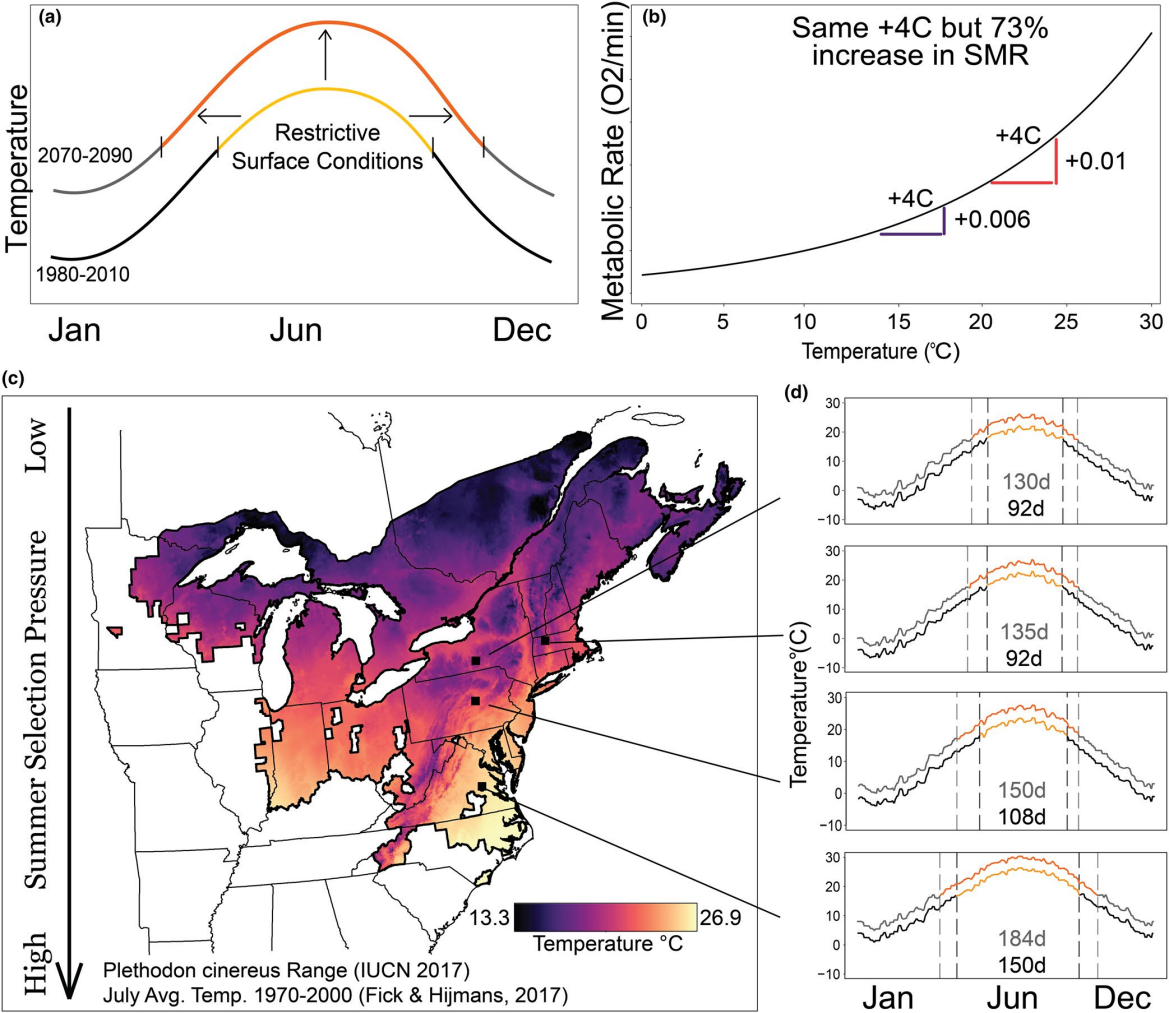
(a) Amphibians can behaviorally avoid extreme temperatures via subterranean refugia, but this comes at the expense of foraging. Under future climates (2070–2090), restrictive summer conditions will get both hotter and longer. (b) Standard metabolic rate is a measure of maintenance energy costs. Metabolic rate scales exponentially with temperature, so warming in the warmest periods (i.e., temperate summers) leads to the highest increases in energy expenses. (c) Our model organism, *Plethodon cinereus*, experiences July average daily temperatures spanning 13.6°C across its range. The populations we study span 6°C, approximately half the thermal range. Mechanisms for saving energy under these conditions are important and likely to be under selection. (d) Terrestrial salamanders generally avoid surface temperatures above 18°C. Days of summer in those populations vary in length (black numbers) and intensity, and all populations expect even longer (grey numbers) and warmer summers in 2070–2090. Managing metabolic rate and during the summer when energy acquisition may be lower is important for maintaining energy balance

Ectotherms comprise the majority of animal biomass and biodiversity on Earth, and their fitness is strongly governed by thermal conditions (Huey & Stevenson, 1979). Increases in temperature directly impact their ecology, including observed extinction or threat of extinction (Sinervo et al., 2010; Urban, 2015). Ectotherm energy budgets drive responses because thermal conditions affect energy acquisition and allocation (Dillon et al., 2010; Guzzo et al., 2017; IPCC, 2013), and previous work in this area has mostly focused on thermophilic invertebrates, reptiles, or fish (Deutsch et al., 2008; Guzzo et al., 2017; Marques et al., 2018; Stoks et al., 2014). In contrast, amphibians, which are subject to increased rates of water loss through permeable skin, often behaviorally avoid warmer temperatures or dry conditions (Peterman & Semlitsch, 2014; Riddel & Sears, 2015). This avoidance could prevent exposure, but it could also reduce foraging (Taub, 1961; Figure 1a). Standard metabolic rate (SMR; using Burton et al., 2011 definition), an indicator of energy spent on maintaining organ system function, increases exponentially as body temperature increases (Clarke, 1993; Savage et al., 2004). This means warmer temperatures are disproportionately more energetically costly (Figure 1b). As a result, amphibian thermal ecology is a complex interplay because energy acquisition is most strongly reduced during the same time maintenance costs would be highest. From this dynamic, we predict that thermal conditions during summer result in the most stressful period for amphibian energy budgets in temperate systems (Naya et al., 2008). Because of the exponential relationship between metabolic rate and temperature, increases in summer temperature over the next century likely pose a threat to amphibian energy balance in temperate climates.

Seasonal temperature regimes are predictable, so thermal acclimation theory predicts that organisms may evolve plasticity (i.e., acclimate) to environmental cues to maximize performance within each season (Angilletta, 2009; Ghalambor, 2006). We define thermal plasticity as the ability of an ectotherm to express multiple phenotypes in response to thermal cues. Thermal plasticity has been observed in a variety of organisms to deal with changing conditions (Rohr et al., 2018). Variation in metabolic rate, and plasticity therein, is important for coping with energy balance and behavior (Burton et al., 2011). Future climate warming is expected to increase the intensity and duration of summer temperatures across the temperate zone, leading to longer and more severe periods of energy stress for amphibians (Allen & Sheridan, 2016). Although amphibians can find microhabitat refugia, typically in the soil, soil surface temperatures are also increasing (Hu & Feng, 2003; Wang et al., 2018). Precipitation is predicted to become more variable in frequency and magnitude and will also affect salamander physiology through avoidance of dry conditions (Hayhoe, Wake, Huntington, et al., 2007; Peterman & Semlitsch, 2014). While salamanders may go to deeper soil depths to find cooler temperatures, this may have consequences on their ability to detect amenable soil surface temperatures for foraging and energy intake (Huey et al., 2021). For salamanders to remain near the surface under longer and hotter summer conditions, seasonal plasticity in metabolic rate may help mitigate higher energy costs.

Past studies show that thermal plasticity in physiological traits such as thermal tolerance may not improve persistence under future climates (Gunderson & Stillman, 2015), but thermal plasticity in physiological rates, such as SMR, has the potential to reduce energetic costs (Seebacher et al., 2014). However, organisms that behaviorally thermoregulate species may not have adapted physiological mechanisms for dealing with stressful thermal conditions because they are behaviorally avoided (Bogert, 1949). This “Bogert effect” is supported in reptiles (Buckley et al., 2015; Gunderson & Stillman, 2015; Sinervo et al., 2010), but it has only recently been considered in amphibian studies (Farallo et al., 2018). If applicable to amphibians, modeling approaches that do not account for intraspecific variation are sufficient for predicting responses to future climate warming. Because plasticity could play an important role in climate adaptation, it is important to determine levels of variation and constraints in thermal plasticity for amphibian physiological rates (Huey et al., 2012; Urban et al., 2014). Predicting responses of amphibians to future changes are complicated by unresolved sources of variation and often because it assumed that a species does not vary over space, time (i.e., plasticity), or across ontogeny (Sinclair et al., 2016; Valladares et al., 2014).

We use the eastern red‐backed salamander (*Plethodon cinereus*) as an amphibian model to describe how SMR and thermal plasticity vary among populations and life stages. *Plethodon cinereus* is a widespread, North American forest‐associated species whose populations are distributed across a range of summer intensities and duration, and thus varying levels of selection pressure to cope with summer conditions (Figure 1c,d). Like many species in the family Plethodontidae, temperature and moisture drive activity patterns, so it is suspected that *P. cinereus* relies on subterranean refugia to find cooler, wetter conditions during the summer (Jaeger, 1980; Peterman & Semlitsch, 2013; Taub, 1961). However, there are likely limits to which elevated temperatures can be avoided. Soil temperatures are largely driven by local climate and air temperature (Paul et al., 2004), and air temperatures largely correlate with soil temperatures up to 20 cm (Islam et al., 2015). If there are not physical access limits, such as impermeable soil depth and geology, there are also potential costs to going too deep, such as not foraging on the forest floor (Huey et al., 2021). In fact, thermal plasticity in metabolic rate to warm conditions in a sister species was recently documented (Riddell et al., 2018a). As soil conditions warm during the summer, salamanders are likely to experience increases in SMR despite thermoregulatory behavior.

We experimentally test for differences among populations, life stages, and thermal plasticity by exposing salamanders from multiple populations to different thermal acclimation regimes. Despite being one of the most widely studied terrestrial salamanders, it is unresolved whether they demonstrate plastic metabolic rates or vary in plasticity across populations or life stages (Feder, 1985; Markle & Kozak, 2018). Despite behavioral thermoregulation, we hypothesize that salamanders will exhibit metabolic downregulation when exposed to summer thermal conditions and will not follow predictions of the Bogert effect. We predict that salamanders from populations with warmer thermal conditions will have the great magnitude in metabolic downregulation. Across life stages we do not expect to find differences because juveniles may have more avenues for developmental plasticity as predicted by theory (Angilletta Jr., 2009).

## 2 METHODS

### 2.1 Site selection and salamander collection

We collected salamanders from four populations in April and May of 2017: Richmond, Virginia; Millmont, Pennsylvania; Ithaca, New York; and Turners Falls, Massachusetts. Mean July nighttime lows varied 6.6°C among populations, which spans half the temperature range this species experiences (Figure 1c). Virginia is the warmest site with the longest summer. New York and Massachusetts have the coolest climates and shortest summer, and Pennsylvania is intermediate in climate and summer length (Figure 1d). For each population, salamanders were collected over 1–2 days by searching underneath rocks and logs. To test for life‐stage differences, 18–20 juveniles and 18–20 adult male salamanders were collected at each site. Females were excluded because we could not control for the varying levels of investment in reproduction, such as size and quantity of eggs. Males were determined by “candling” (Gillette & Peterson, 2001): via visual confirmation of testes and vas deferens through their transparent ventral skin. All but five individuals were the striped morph with the rest being “lead‐backed” morphs. Salamanders were brought to a captive facility at Pennsylvania State University and individually housed inside 11.5 × 11.5 × 5 cm containers within an environmental chamber. We fed salamanders fruit flies daily, *ad libitum*, and maintained unbleached paper towels sprayed with dechlorinated water substrate for hydration during our daily checks on animals. All capture and physiological procedures were approved by IACUC # 47546.

### 2.2 Acclimation and metabolic trials

**FIGURE 2:**
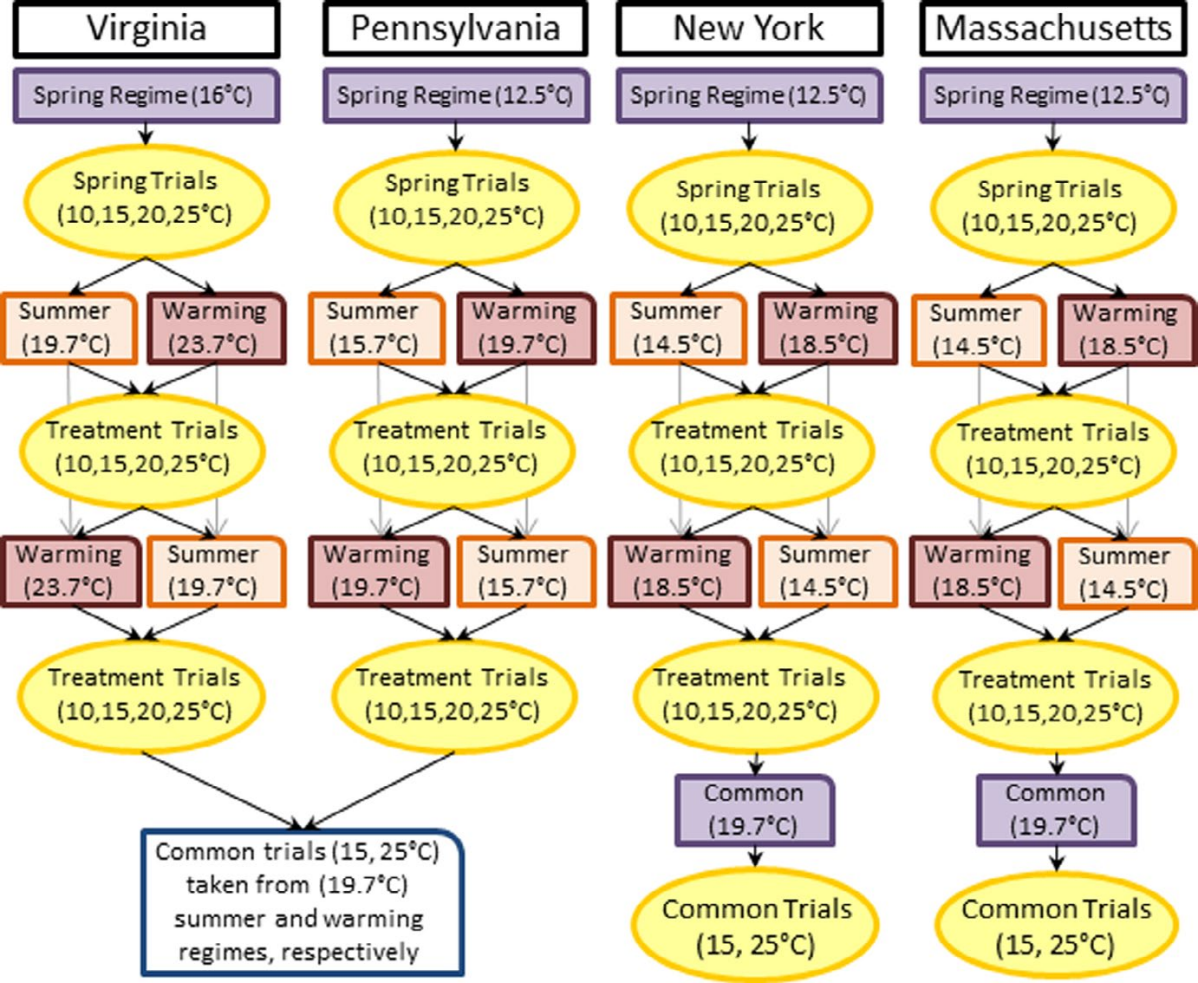
This represents the study design for thermal acclimation regimes salamanders experienced before each round of metabolic trials. For each of the four populations, all salamanders first experienced 10 days of spring temperatures. After 10 days in the spring regime, salamander underwent 4 days of metabolic trials at four different temperatures. Following that half of the animals from each population were randomly assigned to a summer (1980–2010 mean July nighttime temperature) or warming (2070–2090 mean July nighttime temperature) thermal regime. After another round of four temperature trials, treatments were switched. All salamanders had all three thermal acclimation regimes and underwent 12 total metabolic trials. Lastly, Massachusetts and New York had one final thermal regime, 19.7°C, so that metabolic rates could be compared across all four populations under a common temperature. These were only measured at 15°C and 25°C

In total, each salamander had its standard metabolic rate (SMR) measured 12–14 times depending on population. Virginia and Pennsylvania salamanders experienced four trials for each of three thermal acclimation regimes (described below), and Massachusetts and New York salamanders experienced two additional trials under a fourth thermal acclimation regime (Figure 2). The first acclimation regime for all salamanders was “spring.” For this regime, salamanders were housed at the surface soil temperature measured at their capture site so that they would be acclimated to their home spring conditions (Table 1; Figure 2). For three of the populations this happened to be the same temperature. After spring acclimation, salamanders underwent their first four metabolic trials. To generate a thermal reaction norm—mean SMR and the rate it increases with temperature—for salamanders under each acclimation regime, trials were conducted at four temperatures in randomized order: 10°C, 15°C, 20°C, and 25°C. After the spring regime and trials, we randomly assigned half of the salamanders from each population, stratified by life stage (*n* = 18–20), to two thermal regimes: a “summer” treatment that was the 1980–2010 July nighttime climate normal for their home locale (Arguez et al., 2012) or a “warming” treatment which adds 4°C to their local summer temperature to represent the 2070–2090 mean temperature they will experience from climate warming (Hayhoe, Wake, Huntington, et al., 2007). Thermal regimes were specific to each population's local climate so that findings were not confounded by climate transfer distance and represented responses to their home seasonal regimes (Leites et al., 2012). Because different populations had to share environmental chambers, there were slight differences from climate normal for the thermal regimes (on average, <0.48°C off; Table 1). We chose nighttime low temperatures from the 1980–2010 climate normal because soil temperatures are generally cooler than air temperatures during the summer, surface soil temperatures are highly correlated with air temperature (Islam et al., 2015), and because these are the conditions salamanders would be active under. All thermal acclimation regimes for the housing chambers were held at a constant temperature (± 0.02°C). A stronger experimental design would have included temperature variation in thermal regimes to provide more realistic conditions (Bozinovic et al., 2013).

**TABLE 1:** These are the temperatures (°C) used for each thermal acclimation regime salamanders experienced during this study. For comparison to the Summer Thermal Regime, the July Climate Normal is the average low temperature during July 1980–2010 taken from nearby weather stations (Arguez et al., 2012). Salamanders experienced a spring (measured by thermometer at collection site), summer (from 1980–2010 climate normal), and warming thermal regime (summer climate normal +4°C). Housing regimes do not match climate data perfectly because populations needed to share housing (e.g., half of VA salamanders shared housing with half of PA animals, VA receiving its summer thermal regime and PA its warming regime)

	Virginia	Pennsylvania	Massachusetts	New York
Spring Thermal Regime	16	12.5	12.5	12.5
Summer Thermal Regime	19.7	15.7	14.5	14.5
Warming Thermal Regime	23.7	19.7	18.5	18.5
July Climate Normal	20.5	16	13.9	14.3

Salamanders experienced either their local “summer” or “warming” acclimation regimes for 3 weeks before undergoing another four metabolic trials, as described above (Markle & Kozak, 2018). After this round of trials, acclimation regimes were switched, and salamanders were assigned to the summer or warming thermal regime they had not yet experienced (Figure 2). After this round of summer and warming thermal regimes, salamanders had another round of four metabolic trials. At this stage in the study, all salamanders experienced a spring (first acclimation period), local summer, and local warming acclimation thermal regime with the order of summer and warming randomized for individuals to prevent any confounding with time in captivity. From each regime, salamanders had four trials to generate a thermal reaction norm (see analysis section). Salamanders were fed throughout the thermal regime changes and were only fasted 10 days prior to four temperature trials to achieve a post‐absorptive state for measuring SMR (Homyack et al., 2010).

In addition to testing for plasticity using local climate regimes for each population, we also measured metabolic rates for salamanders under a single common thermal regime. This was done to compare SMR among populations. Populations were housed at a common high‐temperature regime (Figure 2; hereafter 19.7°C common regime). This temperature was chosen because it was the closest acclimation regime to the median July normal across the species range (Figure 1c) that most salamanders had already experienced in the study. Note that individuals may have experienced these temperature conditions at different times in the experimental design; salamanders from Virginia (19.7°C summer regime) and Pennsylvania (19.7°C warming regime) experienced this during the treatment acclimation regimes while salamanders from Massachusetts and New York experienced these conditions at the end of the treatment acclimation regimes (Figure 2).

For the metabolic trials, the four temperature trials at 10°C, 15°C, 20°C, and 25°C each occurred on a single day. Temperature order was randomized for each set of trials. These temperatures represent realistic surface and soil temperatures salamanders experience in the summer and spring (Muñoz et al., 2016; Novarro et al., 2018). Because this species is nocturnal, we ran trials during daytime hours between 07:00 a.m. and 06:00 p.m. to minimize activity levels to best characterize SMR. Standard metabolic rate is a conservative estimate of metabolic rate (Burton et al., 2011). Mass of individual salamanders was measured before and after every trial. For comparisons of standard metabolic rate at the 19.7°C common acclimation regimes, metabolic trials were only done at 15°C and 25°C to minimize extra trials salamanders experience, to characterize the Q10 response for the warmer trial temperatures, and because preliminary data showed the linear relationship was consistent from 10°C to 20°C as it was 15°C to 25°C.

Salamander SMR was measured using stop‐flow respirometry following established manual bolus integration calculations (Lighton, 2008). Salamanders were individually loaded into 60 mL syringes and flushed for 60 s with dry, carbonless air, and then sealed. To ensure measurable oxygen consumption, syringes were sealed for 5 h (10°C and 15°C), 4 h (20°C), and 3 h (25°C). Upon completion, 30 mL of syringe air was injected into the stop‐flow system, and average oxygen consumption was measured (Oxzilla II, Sable Systems,). Flow rates were 80 ml × min^−1^ (Mass Flow Controller 5850E, Brooks Instrument), and prior to sensor entry, CO_2_ and H_2_O were scrubbed from the incoming air with Ascarite II (Thomas Scientific) and magnesium perchlorate, respectively. Raw data were recorded using Expedata software (Sable Systems) and processed in program R (R Core Team, 2018).

### 2.3 Analysis

To test our hypotheses, we wanted to compare the reaction norms within each population to see if plasticity was exhibited in SMR among thermal acclimation regimes. We also wanted to compare reaction norms among populations from the 19.7°C common regime. To do this we ran five separate models to investigate patterns in salamander SMR. We used log(SMR) as the response variable for each individual salamander to account for exponential relationships with body mass and temperature. We modeled log body mass (log(g)) and day of trial (1–4) as fixed effects. Repeated measures were modeled using a random intercept for individual. Random slopes were not supported in our model selection process (Appendix S1). We considered body mass, day of trial, and individual variation as nuisance variables. Other nuisance parameters were considered but were removed because of high variance inflation factors (Appendix S1: Table S2). The first model analyzed differences among populations (fixed effect) under the 19.7°C common regime. The other four models tested for plasticity and analyzed each population separately with thermal acclimation regime (spring, summer, and warming) as a fixed effect. To determine additive versus interactive relationships between fixed effects (population or thermal acclimation regime with temperature), we used Akaike's Information Criteria in a maximum likelihood framework to determine top models (Appendix S1: Table S3–S6). Top models were analyzed using a restrictive maximum likelihood estimator in package “lme4” (Bates et al., 2015) to run a mixed‐effects Gaussian regression using function “lmer.” With log transformation, residuals were homoscedastic and passed visual assessment of normality via Q‐Q plots. Temperature predictor variables were centered and standardized by standard deviation to aid in computation. We tested for differences in mean SMR, which were the intercept of the model and represented metabolic rate per fixed effect at the average trial temperature and body mass. We also looked for differences in thermal sensitivity, the slope term that represented the increase in SMR by increase in temperature for each fixed effect.

### 2.4 Data repository

All data and program R scripts can be found at Data Dryad (https://doi.org/10.5061/dryad.931zcrjm5).

## 3 RESULTS

We used standard metabolic rate (SMR) from 1720 trials on 72 juvenile and 67 adult male salamanders from four populations. Five adult males had died in captivity soon after arrival. No cause was identified from autopsy. Juveniles had an average body mass of 0.50 g (± 0.14 SD) and adults 0.96 g (± 0.19 SD). During trials, salamanders lost on average 3.3% of their body mass, but they frequently regained mass lost within 24 h via rehydration. Across 63 days of captivity, juveniles lost on average 0.025g (4.7% of average juvenile body mass), and adult males lost on average 0.069g (7.6% of average adult body mass). After controlling for mass and individual variability, estimated average SMR thermal sensitivity—the rate at which log(SMR) increases with log(Temperature)—of populations ranged from 1.5 (± 0.04 SE) to 1.7 (± 0.03 SE). Scaling of SMR with body mass also varied across populations, with the mass scaling exponent ranging from 0.53 (± 0.07 SE) to 0.86 (± 0.06 SE; Appendix S1: Figure S1). These mass scaling exponents differ from the expected value of 0.66 predicted by the metabolic theory of ecology (Brown et al., 2004).

**FIGURE 3:**
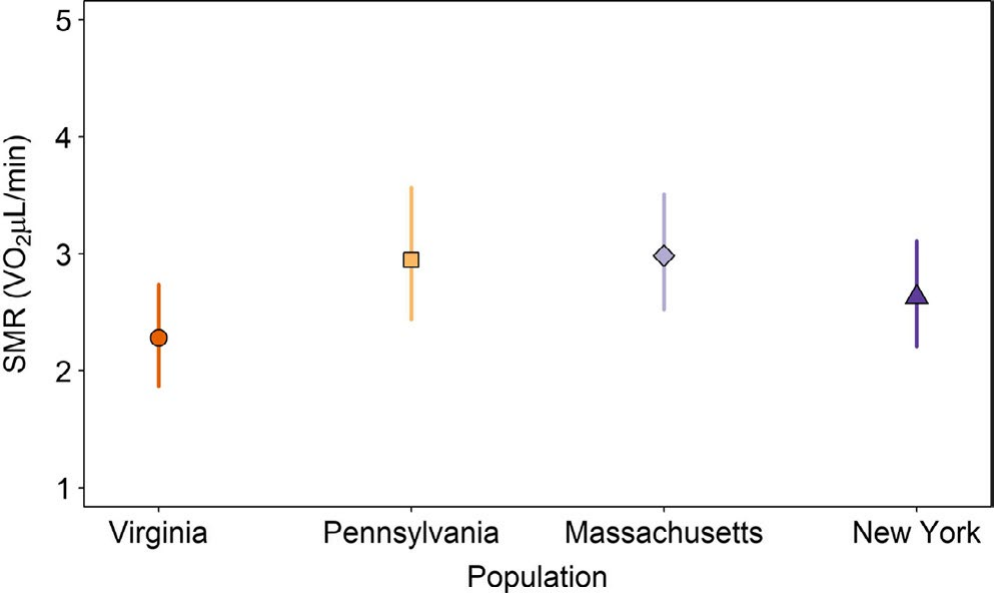
At the 19.7°C common thermal acclimation regime, all populations exhibited variation in mean standard metabolic rate. Virginia and New York had the lowest mean standard metabolic rate, but all populations had similar means and were not statistically significant from one another. Only the mean standard metabolic rates are visualized because thermal sensitivity (temperature by population slope interactions) was not supported in the final model

We determined whether SMR varied among populations under the 19.7˚C common regime to determine if populations had similar metabolic rates after being acclimated to similar conditions. Changes in mean SMR (intercept), not thermal sensitivity (slope parameter with temperature), best explained differences among populations (Appendix S1: Table S6). Under these conditions, Virginia had the lowest mean SMR, but all populations had overlapping 95% confidence intervals, indicating overall similar SMR (Figure 3). Populations did vary in their allometric scaling of mass and SMR (Appendix S1: Figures S1). The amount of variation explained by fixed effects in the model (log(mass), population, and the order of the individual trials) was *r* ^2^ = 0.73. For the effect sizes for each parameter, please see Appendix S1.

**FIGURE 4:**
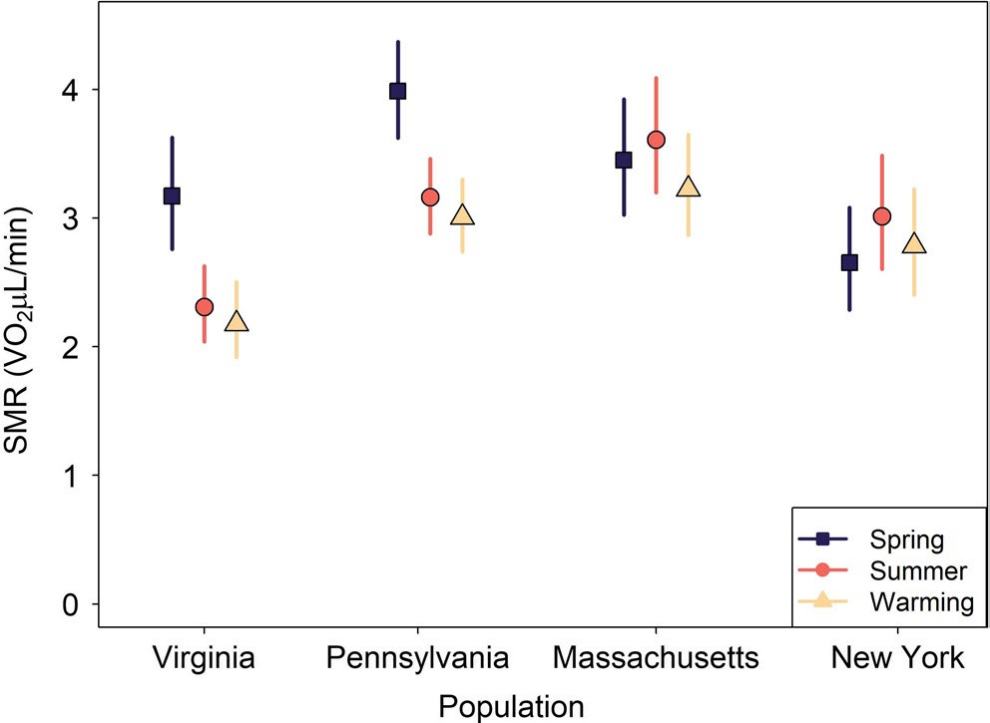
Plasticity in mean standard metabolic rate (SMR; shapes; µL O_2_/min) varied among populations as exhibited by changes after exposure to thermal acclimation regimes (see legend). Both Virginia and Pennsylvania had statistically significant reductions in SMR in response to summer and warming thermal regimes compared to spring regimes (VA −27.2%, PA −20%), but Massachusetts and New York exhibited no plasticity in SMR. No populations exhibited plasticity between current summers and future warming, indicating a lack of plasticity to future conditions. Only the mean standard metabolic rates (intercept terms) are visualized because they represented the strongest statistical finding

Models testing for plasticity under thermal acclimation regimes showed that variation within populations existed. Treatment thermal acclimation regimes were a significant predictor of mean SMR (intercept) for salamander populations in the two warmer climates (Virginia and Pennsylvania; Figure 4; Table 2; significance determined by 95% confidence intervals is not overlapping). Salamanders from warmer sites reduced mean SMR after exposure to summer regimes compared to spring regimes. The greatest reduction in mean SMR occurred in Virginia salamanders (−27.2%) followed by Pennsylvania salamanders (−20.6%; Figure 4; Table 2). The two cooler climate populations did not show any significant change in mean SMR (Figure 4). Of the four populations, Massachusetts was the only population to include an interaction between thermal acclimation regime and SMR thermal sensitivity (slope) in its final model. Compared to the spring acclimation, mean summer thermal sensitivity decreased slightly with increasing temperatures (−0.08, [−0.159, −0.005] 95% CI). All populations exhibited less than 10% individual variation in their random intercepts. These models explained a high level of variation in metabolic rate: Virginia *r* ^2^ = 0.55, Pennsylvania *r* ^2^ = 0.79, Massachusetts *r* ^2^ = 0.76, and New York *r* ^2^ = 0.59. All regression coefficients can be found in Appendix S1, Table S7.

**TABLE 2:** Mean estimated standard metabolic rate (SMR; µL O_2_/min), with 95% bootstrapped confidence intervals, for a typical adult male from each population. Mean SMR vary by thermal regimes (spring, summer, and warming). Metabolism was downregulated between spring and summer by Virginia (VA) and Pennsylvania (PA) salamanders, but not the Massachusetts (MA) or New York (NY) populations. Warming mean SMR was lower than summer, but confidence intervals overlapped, indicating no clear differences between summer and warming SMR

	Spring SMR	Summer SMR	Warming SMR	Spring vs. Summer	Summer vs. Warming
VA	3.17 (2.76, 3.62)	2.31 (2.04, 2.63)	2.18 (1.92, 2.5)	−27.2%	−5.7%
PA	3.99 (3.62, 4.37)	3.16 (2.88, 3.46)	3 (2.74, 3.3)	−20.6%	−5.0%
MA	3.45 (3.03, 3.92)	3.61 (3.2, 4.09)	3.22 (2.87, 3.65)	+4.6%	−10.7%
NY	2.64 (2.29, 3.08)	3 (2.61, 3.49)	2.77 (2.4, 3.23)	+13.6%	−7.6%

We did not find consistent support for differences between life stages in their SMR. Juvenile salamanders showed similar plasticity as adult males in all populations except for Pennsylvania. Juvenile salamanders from Pennsylvania had a slight (0.242) but statistically significant lower intercept (i.e., average SMR) than adult male salamanders. In response to summer thermal regimes, Pennsylvania juvenile salamanders exhibited a slightly smaller reduction in SMR compared to adult salamanders (Appendix S1: Table S7). Separate from thermal relationships, Massachusetts and Pennsylvania juvenile salamander had lower intercepts for the mass relationships, indicating that metabolic rate increases more slowly as body mass increases than in adults (Appendix S1: Figure S1).

## 4 DISCUSSION

We find that *P. cinereus* can exhibit plasticity in standard metabolic rate (SMR) in response to seasonal thermal cues. Perhaps unsurprisingly for a limited dispersal species with a large geographic range, capacity for plastic response was not consistent among populations. Across the populations of *P. cinereus* we studied, plasticity in mean SMR appears to be present only in the populations from the warmer climates. Documenting this phenomenon suggests thermal plasticity functionally reduces energy costs during stressful summer conditions and could be adaptive. Without a thermally plastic response, populations in warmer parts of the species’ range would experience a significantly higher maintenance energy costs which could infer a mismatch between energy requirements and foraging opportunities. Our findings suggest that plasticity in SMR in salamanders from warmer climates, but not cooler, may be a mechanism for reducing maintenance energy costs over the summer. However, when exposed to predicted summer thermal conditions expected in 2070–2090 (i.e., “warming” regime), all populations demonstrated no further plasticity in SMR. This indicates that there are limits to the adaptive capacity plasticity infers to thermal regimes not experienced before.

Our findings help inform which ecological and evolutionary theories may apply to terrestrial amphibians. Our findings of plasticity and among‐population variation therein lend no support for the Bogert effect in this salamander species (Bogert, 1949; Farallo et al., 2018). Behavioral thermoregulation, which is widespread in this species, did not preclude physiological adaptations in SMR. Our finding reveals that some salamander populations follow predictions from thermal acclimation theory by expressing plasticity in SMR in response to seasonal thermal cues (Angilletta, 2009). However, seasonality, and thus plasticity, is hypothesized to be more pronounced in cooler parts of the *P. cinereus* range (Ghalambor, 2006). Our study showed that it was populations from the two warmer climates that exhibited the strongest thermal plasticity. Deutsch et al. (2008) demonstrated that the warmest climates (i.e., tropics) drive susceptibility of ectotherms to a negative energy balance. For temperate zone *Plethodon* salamanders, summer represents the period of warmest conditions. Terrestrial salamander body temperatures rapidly reach equilibrium with the surrounding environment (Lunghi et al., 2016). Given behavioral avoidance of warmer temperatures in *P. cinereus* (Muñoz et al., 2016), our findings may lend support that the warm conditions during the summer constitute a significant pressure on salamander energy budgets. A further step in improving our understanding is to include variation in acclimation temperatures to better reflect natural conditions (Terblanche et al., 2010).

There is currently a renewed interest in characterizing terrestrial salamander physiology (Gifford, 2016) because they are often thought as indicator species of forest habitats with important roles in ecosystem processes (Burton & Likens, 1975; Hocking & Babbitt, 2014; Welsh & Droege, 2001). A study recently suggested that *P. cinereus* does not have plasticity in metabolic rate or any variation among populations (Markle & Kozak, 2018); however, they had a small number of salamanders from each population, and they did not consider a reduction in SMR after exposure to warmer temperatures to be indicative of plasticity. Past investigations in salamander acclimation found that four species of Plethodontidae, including *P*. *cinereus*, had decreased metabolic rates after being exposed to a 17.5°C thermal regime compared to a 4–5°C regime, which was believed to be an adaptive energy saving mechanism (Feder, 1985). Plastic reduction in SMR, in combination with behavior, has been shown to greatly increase the resistance of a different terrestrial salamander species to extinction (Riddell et al., 2018a). To our knowledge, our study is the first to provide evidence of geographic variation in plasticity of metabolic rate for terrestrial salamanders. Because of our documentation of plasticity, future studies should include multiple common acclimation thermal regimes when testing for differences among populations to capture consistency more adequately in salamander's performance in metabolic trials. We affirm other studies that argue that biophysical and species distribution approaches cannot assume that all populations share the same responses (Cotto et al., 2017; Franklin, 2010; Urban et al., 2016).

The role of thermal plasticity in aiding species persistence under climate change is unresolved (Canale & Henry, 2010; Metcalfe & Norin, 2019). There is evidence that plasticity in physiological traits can both increase and decrease adaptive capacity of a species (Gomez‐Mestre & Jovani, 2013; Gunderson & Stillman, 2015; Kingsolver & Buckley, 2017; Oostra et al., 2018; Seebacher et al., 2014). It has been argued that a better understanding of limits to plasticity is needed to clarify its adaptive potential (Metcalfe & Norin, 2019). It is challenging to determine if plasticity in metabolic rate confers a selective benefit because the adaptive capacity of a high or low SMR is context dependent (Burton et al., 2011). For instance, high metabolic rates are beneficial for increasing resource acquisition and performance in energetically expensive behaviors, but not when resources are scarce or when maintenance energy costs need to be minimized (Burton et al., 2011; Metcalfe & Norin, 2019). Low metabolic rates are beneficial for saving energy, but they can put organisms at a disadvantage when active behaviors are needed (Burton et al., 2011).

Under current climate conditions, we provide conclusive evidence that salamanders in some populations have reduced SMR after exposure to summer temperature cues. We argue this is adaptive because this plasticity was evident in the two populations with the warmer and longer summers, a period when salamanders have reduced foraging ability and higher maintenance energy costs. Under these conditions, our observed 20.6%–27.2% reduction in metabolic rate would lead to a larger savings in maintenance energy costs. For populations in the warm edges of the species range, thermal plasticity in SMR could aid population persistence, as has been found for another terrestrial salamander species (Riddell et al., 2018a). This plasticity is likely adaptive in reducing maintenance energy costs (Burton et al., 2011; Christian et al., 1999; Metcalfe & Norin, 2019). Reduction in energy metabolism has also been argued as adaptive for other ectotherms (Artacho & Nespolo, 2009). Under future climate conditions, we found all populations in our study lacked thermal plasticity to further reduce metabolic rate. This supports theory which predicts that organisms will not evolve plasticity to conditions they have not yet experienced (Angilletta, 2009). Future avenues of research should determine the degree to which the plasticity we documented, and lack of plasticity to future climate thermal cues, is expected adaptive or maladaptive for whole salamander energy budgets.

We found mixed evidence for differences in SMR between life stages. Juveniles from Pennsylvania exhibited slightly lower levels of plasticity compared to adult males (Appendix S1). By having slightly less plastic SMR, either juveniles in Pennsylvania have increased susceptibility to warmer temperatures, or other forms of plasticity (e.g., developmental) may contribute to resilience (Burggren, 2018). For instance, both Pennsylvania and Massachusetts juveniles had lower body mass–SMR scaling relationships (Appendix S1). Following the allometric scaling of metabolic rate with body mass (Brown et al., 2004), having a lower scaling relationship means being smaller and not investing energy into reproduction lowers costs of warm temperatures, but future analyses should disentangle the sensitivity of different life stages in regard to energy budgets. For instance, in fishes, body size varied counter to temperature, suggesting smaller body mass offsets high maintenance energy costs from warmer temperatures (Moffett et al., 2018). Temperature–size relationships in amphibians are contradictory and unresolved (Ashton, 2002; Peterman et al., 2016). Importantly, our findings show that similar levels of plasticity can generally be assumed between juveniles and adult males. Our inference could be improved by including female salamanders; however, accounting for reproductive status is challenging (Finkler, 2006).

Using SMR to predict population outcomes remains challenging (Buckley et al., 2014), mainly because the fitness advantage of high or low SMR is context dependent (Burton et al., 2011). However, energy allocated to maintenance cannot be spent on growth or reproduction, meaning changes in SMR are strong predictors of changes in life‐history traits (Le Lann et al., 2011; Pettersen et al., 2015, 2016). Across animal taxa, experimental warming reduced maximum body size, spurred earlier reproduction, and reduced longevity (Bestion et al., 2015; Ohlberger, 2013; Winkler et al., 2002). Energy assimilation is also expected to decrease at warmer temperatures (Fontaine et al., 2018). To persist under future climates, salamanders may need to increase energy acquisition via phenological shifts to maintain current allocation. Without changes to energy acquisition, populations could experience reduced maximum body size, reproduce at smaller sizes, or have reduced fecundity. Resolving how life‐history traits are affected by temperature changes is necessary to clarify the impacts of higher maintenance energy costs on population persistence under future climates (Moffett et al., 2018).

We identified previously unknown geographic variation in plasticity in terrestrial salamanders (Riddell et al., 2018b) and show that plasticity in SMR responds to current summer seasonal cues as a potential energy‐saving mechanism. Our findings also identify limits to plasticity, suggesting that current levels of plasticity in SMR may not respond to future climate conditions (Metcalfe & Norin, 2019). Our findings reveal fundamental patterns in physiological rates that can be used to parameterize biophysical climate models (Kearney & Porter, 2009; Urban et al., 2016) and point to necessary life‐history and demographic data needed to resolve persistence or extinction of terrestrial salamanders under climate change.

## CONFLICT OF INTEREST

We declare no conflict of interest regarding patent or stock ownership, membership of a company board of directors, membership of an advisory board or committee for a company, and consultancy for or receipt of speaker's fees from a company.

## AUTHOR CONTRIBUTION

**David Jonathan Muñoz:** Conceptualization (lead); Data curation (lead); Formal analysis (lead); Funding acquisition (lead); Investigation (lead); Methodology (equal); Project administration (lead); Resources (supporting); Software (lead); Validation (lead); Visualization (lead); Writing – original draft (lead); Writing – review & editing (lead). **David A W Miller:** Conceptualization (equal); Formal analysis (supporting); Funding acquisition (lead); Investigation (supporting); Methodology (supporting); Supervision (supporting); Validation (equal); Visualization (equal); Writing – original draft (supporting); Writing – review & editing (supporting). **Rudolf Schilder:** Formal analysis (supporting); Investigation (equal); Methodology (equal); Resources (equal); Software (equal); Supervision (equal); Validation (equal); Writing – original draft (supporting); Writing – review & editing (equal). **Evan H Campbell Grant:** Conceptualization (equal); Funding acquisition (lead); Investigation (supporting); Resources (supporting); Writing – original draft (supporting); Writing – review & editing (supporting).

## Supporting information

Appendix S1

## Data Availability

The data supporting the results are archived in Data Dryad Repository (https://doi.org/10.5061/dryad.931zcrjm5).

## References

[ece38433-bib-0001] Allen, M. J. , & Sheridan, S. C. (2016). Evaluating changes in season length, onset, and end dates across the United States (1948–2012). International Journal of Climatology, 36, 1268–1277. 10.1002/joc.4422

[ece38433-bib-0002] Angilletta, M. J. Jr (2009). Thermal acclimation. In: Thermal adaptation: a theoretical and empirical synthesis (pp. 126–156). Oxford University Press.

[ece38433-bib-0003] Angilletta, M. J. (2009). Thermal adaptation: a theoretical and empirical synthesis. Oxford University Press.

[ece38433-bib-0004] Arguez, A. , Durre, I. , Applequist, S. , Vose, R. S. , Squires, M. F. , Yin, X. , Heim, R. R. , & Owen, T. W. (2012). NOAA’s 1981–2010 U.S. climate normals. Bulletin of the American Meteorological Society, 10.1175/BAMS-D-11-00197.1

[ece38433-bib-0005] Ashton, K. G. (2002). Do amphibians follow Bergmann’s rule ? Canadian Journal of Zoology, 80, 708–716. 10.1139/Z02-049

[ece38433-bib-0091] Artacho, P. , & Nespolo, R. F. (2009). Natural selection reduces energy metabolism in the garden snail, Helix aspersa (Cornu aspersum). Evolution, 63, 1044–1050. 19236475 10.1111/j.1558-5646.2008.00603.x

[ece38433-bib-0006] Bates, D. , Maechler, M. , Bolker, B. , & Walker, S. (2015). Fitting linear mixed‐effects models using lme4. Journal of Statistical Software, 67, 1–48.

[ece38433-bib-0007] Bestion, E. , Teyssier, A. , Richard, M. , Clobert, J. , & Cote, J. (2015). Live fast, die young: experimental evidence of population extinction risk due to climate change. PLoS Biology, 13, 1–19. 10.1371/journal.pbio.1002281 PMC4621050 26501958

[ece38433-bib-0008] Bogert, C. M. (1949). Thermoregulation in reptiles, a factor in evolution. Evolution, 3, 195–211. 10.1111/j.1558-5646.1949.tb00021.x 18138377

[ece38433-bib-0009] Bozinovic, F. , Catalan, T. P. , Estay, S. , & Sabat, P. (2013). Acclimation to daily thermal variability drives the metabolic performance curve. Evolutionary Ecology Research, 15, 579–587.

[ece38433-bib-0010] Brown, J. , Gillooly, J. , Allen, A. , Savage, V. , & West, G. (2004). Toward a metabolic theory of ecology. Ecology, 85, 1771–1789. 10.1890/03-9000

[ece38433-bib-0011] Buckley, L. B. (2008). Linking traits to energetics and population dynamics to predict lizard ranges in changing environments. American Naturalist, 171, E1–E19. 10.1086/523949 18171140

[ece38433-bib-0012] Buckley, L. B. , Ehrenberger, J. C. , & Angilletta, M. J. (2015). Thermoregulatory behaviour limits local adaptation of thermal niches and confers sensitivity to climate change. Functional Ecology, 29, 1038–1047. 10.1111/1365-2435.12406

[ece38433-bib-0013] Buckley, L. B. , Nufio, C. R. , & Kingsolver, J. G. (2014). Phenotypic clines, energy balances and ecological responses to climate change. Journal of Animal Ecology, 83, 41–50. 10.1111/1365-2656.12083 23662736

[ece38433-bib-0014] Burggren, W. (2018). Developmental phenotypic plasticity helps bridge stochastic weather events associated with climate change. Journal of Experimental Biology, 221, jeb161984. 10.1242/jeb.161984 29748332

[ece38433-bib-0015] Burton, T. , Killen, S. S. , Armstrong, J. D. , & Metcalfe, N. B. (2011). What causes intraspecific variation in resting metabolic rate and what are its ecological consequences? Proc. R. Soc, 278, 3465–3473. 10.1098/rspb.2011.1778 PMC3189380 21957133

[ece38433-bib-0016] Burton, T. , & Likens, G. (1975). Salamander populations and biomass in the Hubbard Brook experimental forest, New Hampshire. Copeia, 1975, 541–546. 10.2307/1443655

[ece38433-bib-0017] Canale, C. I. , & Henry, P. Y. (2010). Adaptive phenotypic plasticity and resilience of vertebrates to increasing climatic unpredictability. Climate Research, 43, 135–147. 10.3354/cr00897

[ece38433-bib-0018] Christian, K. A. , Bedford, G. S. , & Schultz, T. J. (1999). Energetic consequences of metabolic depression in tropical and temperate‐zone lizards. Australian Journal of Zoology, 47, 133–141. 10.1071/ZO98061

[ece38433-bib-0019] Clarke, A. (1993). Seasonal acclimatization and latitudinal compensation in metabolism: do they exist? Functional Ecology, 7, 139–149. 10.2307/2389880

[ece38433-bib-0020] Cotto, O. , Wessely, J. , Georges, D. , Klonner, G. , Schmid, M. , Dullinger, S. , Thuiller, W. , & Guillaume, F. (2017). A dynamic eco‐evolutionary model predicts slow response of alpine plants to climate warming. Nature Communications, 8, 15399. 10.1038/ncomms15399 PMC5424169 28474676

[ece38433-bib-0021] Des Roches, S. , Post, D. M. , Turley, N. E. , Bailey, J. K. , Hendry, A. P. , Kinnison, M. T. , Schweitzer, J. A. , & Palkovacs, E. P. (2018). The ecological importance of intraspecific variation. Nature Ecology & Evolution, 2, 57–64. 10.1038/s41559-017-0402-5 29203921

[ece38433-bib-0022] Deutsch, C. A. , Tewksbury, J. J. , Huey, R. B. , Sheldon, K. S. , Ghalambor, C. K. , Haak, D. C. , & Martin, P. R. (2008). Impacts of climate warming on terrestrial ectotherms across latitude. Proceedings of the National Academy of Sciences, 105, 6668–6672. 10.1073/pnas.0709472105 PMC2373333 18458348

[ece38433-bib-0023] Dillon, M. E. , Wang, G. , & Huey, R. B. (2010). Global metabolic impacts of recent climate warming. Nature, 467, 704–706. 10.1038/nature09407 20930843

[ece38433-bib-0024] Farallo, V. R. , Wier, R. , & Miles, D. B. (2018). The Bogert effect revisited: Salamander regulatory behaviors are differently constrained by time and space. Ecology and Evolution, 8, 11522–11532. 10.1002/ece3.4590 30598753 PMC6303756

[ece38433-bib-0025] Feder, M. E. (1985). Acclimation to constant and variable temperatures in Plethodontid salamanders: rates of oxygen consumption. Comparative Biochemistry and Physiology, A: Comparative Physiology, 81, 673–682. 10.1016/0300-9629(85)91046-1 2863058

[ece38433-bib-0026] Fick, S. E. , & Hijmans, R. J. (2017). WorldClim 2: new 1‐km spatial resolution climate surfaces for global land areas. International Journal of Climatology, 37(12), 4302–4315. 10.1002/joc.5086

[ece38433-bib-0027] Finkler, M. S. (2006). Effects of temperature, sex, and gravidity on the metabolism of small‐mouthed salamanders, *Ambystoma texanum*, during the reproductive season. Journal of Herpetology, 40, 103–106. 10.1670/104-05N.1

[ece38433-bib-0028] Fontaine, S. S. , Novarro, A. J. , & Kohl, K. D. (2018). Environmental temperature alters the digestive performance and gut microbiota of a terrestrial amphibian. Journal of Experimental Biology, 221, 10.1242/jeb.187559 30171093

[ece38433-bib-0029] Franklin, J. (2010). Moving beyond static species distribution models in support of conservation biogeography. Diversity and Distributions, 16, 321–330. 10.1111/j.1472-4642.2010.00641.x

[ece38433-bib-0030] Ghalambor, C. K. (2006). Are mountain passes higher in the tropics? Janzen’s hypothesis revisited. Integrative and Comparative Biology, 46, 5–17. 10.1093/icb/icj003 21672718

[ece38433-bib-0031] Gifford, M. E. (2016). Physiology of Plethodontid salamanders: a call for increased efforts. Copeia, 2016, 42–51. 10.1643/OT-14-223

[ece38433-bib-0032] Gillette, J. R. , & Peterson, M. G. (2001). The benefits of transparency : Candling as a simple method for determining sex in red‐backed salamanders (*Plethodon cinereus*). Herpetological Review, 32, 233–235.

[ece38433-bib-0033] Gomez‐Mestre, I. , & Jovani, R. (2013). A heuristic model on the role of plasticity in adaptive evolution: plasticity increases adaptation, population viability and genetic variation. Proceedings of the Royal Society B, 280, 20131869. 10.1098/rspb.2013.1869 24068357 PMC3790484

[ece38433-bib-0034] Gunderson, A. R. , & Stillman, J. H. (2015). Plasticity in thermal tolerance has limited potential to buffer ectotherms from global warming. Proceedings of the Royal Society B, 282, 20150401. 10.1098/rspb.2015.0401 25994676 PMC4455808

[ece38433-bib-0035] Guzzo, M. M. , Blanchfield, P. J. , & Rennie, M. D. (2017). Behavioral responses to annual temperature variation alter the dominant energy pathway, growth, and condition of a cold‐water predator. Proceedings of the National Academy of Sciences, 114, 9912–9917. 10.1073/pnas.1702584114 PMC5604001 28808011

[ece38433-bib-0036] Hayhoe, K. , Wake, C. , Anderson, B. , Liang, X.‐Z. , Maurer, E. , Zhu, J. , Bradbury, J. , DeGaetano, A. , Stoner, A. M. , & Wuebbles, D. (2007). Regional climate change projections for the Northeast USA. Mitigation and Adaptation Strategies for Global Change, 13, 425–436. 10.1007/s11027-007-9133-2

[ece38433-bib-0037] Hayhoe, K. , Wake, C. P. , Huntington, T. G. , Luo, L. , Schwartz, M. D. , Sheffield, J. , Wood, E. , Anderson, B. , Bradbury, J. , DeGaetano, A. , Troy, T. J. , & Wolfe, D. (2007). Past and future changes in climate and hydrological indicators in the US Northeast. Climate Dynamics, 28, 381–407. 10.1007/s00382-006-0187-8

[ece38433-bib-0038] Hocking, D. J. , & Babbitt, K. J. (2014). Effects of red‐backed salamanders on ecosystem functions. PLoS One, 9, e86854. 10.1371/journal.pone.0086854 24466269 PMC3899337

[ece38433-bib-0039] Homyack, J. A. , Haas, C. A. , & Hopkins, W. A. (2010). Influence of temperature and body mass on standard metabolic rate of eastern red‐backed salamanders (*Plethodon cinereus*). Journal of Thermal Biology, 35, 143–146. 10.1016/j.jtherbio.2010.01.006

[ece38433-bib-0040] Hu, Q. , & Feng, S. (2003). A daily soil temperature dataset and soil temperature climatology of the contiguous United States. Journal of Applied Meteorology, 42, 1139–1156. 10.1175/1520-0450(2003)042<1139:ADSTDA>2.0.CO;2

[ece38433-bib-0041] Huey, R. B. , Kearney, M. R. , Krockenberger, A. , Holtum, J. A. M. , Jess, M. , & Williams, S. E. (2012). Predicting organismal vulnerability to climate warming: roles of behaviour, physiology and adaptation. Philosophical Transactions of the Royal Society B: Biological Sciences, 367, 1665–1679. 10.1098/rstb.2012.0005 PMC3350654 22566674

[ece38433-bib-0042] Huey, R. B. , Ma, L. , Levy, O. , & Kearney, M. R. (2021). Three questions about the eco‐physiology of overwintering underground. Ecology Letters, 24, 170–185. 10.1111/ele.13636 33289263

[ece38433-bib-0043] Huey, R. B. , & Stevenson, R. D. (1979). Integrating thermal physiology and ecology of ectotherms: a discussion of approaches. Integrative and Comparative Biology, 19, 357–366. 10.1093/icb/19.1.357

[ece38433-bib-0044] IPCC . (2013). Climate change 2013: the physical science basis. Contrib. Work. Gr. I to Fifth Assess. Rep. Intergov. Panel Clim. Chang. [Stocker, Thomas F. Qin, Dahe Plattner, Gian‐Kasper Tignor, Melinda M.B. Allen, Simon K. Boschung, Judith Nauels, Alexander Xia, Yu Bex, Vincen]. 10.1038/446727a

[ece38433-bib-0045] Islam, K. I. , Khan, A. , & Islam, T. (2015). Correlation between atmospheric temperature and soil temperature: a case study for Dhaka, Bangladesh. Atmospheric and Climate Sciences, 5, 200–208. 10.4236/acs.2015.53014

[ece38433-bib-0046] Jaeger, R. G. (1980). Microhabitats of a terrestrial forest salamander. Copeia, 1980, 265–268. 10.2307/1444003

[ece38433-bib-0047] Kearney, M. , & Porter, W. (2009). Mechanistic niche modelling: combining physiological and spatial data to predict species’ ranges. Ecology Letters, 12, 334–350. 10.1111/j.1461-0248.2008.01277.x 19292794

[ece38433-bib-0048] Kingsolver, J. G. , & Buckley, L. B. (2017). Evolution of plasticity and adaptive responses to climate change along climate gradients. Proceedings of the Royal Society B, 284, 20170386. 10.1098/rspb.2017.0386 28814652 PMC5563792

[ece38433-bib-0049] Le Lann, C. , Wardziak, T. , van Baaren, J. , & van Alphen, J. J. M. (2011). Thermal plasticity of metabolic rates linked to life‐history traits and foraging behaviour in a parasitic wasp. Functional Ecology, 25, 641–651. 10.1111/j.1365-2435.2010.01813.x

[ece38433-bib-0050] Leites, L. P. , Robinson, A. P. , Rehfeldt, G. E. , Marshall, J. D. , & Crookston, N. L. (2012). Height‐growth response to climatic changes differs among populations of Douglas‐fir: A novel analysis of historic data. Ecological Applications, 22, 154–165. 10.1890/11-0150.1 22471081

[ece38433-bib-0051] Lighton, J. R. (2008). Measuring metabolic rates. Oxford University Press. 10.1017/CBO9781107415324.004

[ece38433-bib-0052] Lunghi, E. , Manenti, R. , Canciani, G. , Scarì, G. , Pennati, R. , & Ficetola, G. F. (2016). Thermal equilibrium and temperature differences among body regions in European Plethodontid salamanders. Journal of Thermal Biology, 60, 79–85. 10.1016/j.jtherbio.2016.06.010 27503719

[ece38433-bib-0053] Markle, T. M. , & Kozak, K. H. (2018). Low acclimation capacity of narrow‐ranging thermal specialists exposes susceptibility to global climate change. Ecology and Evolution, 8, 4644–4656. 10.1002/ece3.4006 29760904 PMC5938462

[ece38433-bib-0054] Marques, G. M. , Augustine, S. , Lika, K. , Pecquerie, L. , Domingos, T. , & Kooijman, S. A. L. M. (2018). The AmP project: Comparing species on the basis of dynamic energy budget parameters. PLoS Computational Biology, 14, 1–23. 10.1371/journal.pcbi.1006100 PMC5962104 29742099

[ece38433-bib-0055] McCain, C. , Szewczyk, T. , & Bracy Knight, K. (2016). Population variability complicates the accurate detection of climate change responses. Global Change Biology, 22, 2081–2093. 10.1111/gcb.13211 26725404

[ece38433-bib-0056] Metcalfe, N. B. , & Norin, T. (2019). Ecological and evolutionary consequences of metabolic rate plasticity in response to environmental change. Philosophical Transactions of the Royal Society B: Biological Sciences, 374(1768), 1–9. 10.1098/rstb.2018.0180 PMC6365862 30966964

[ece38433-bib-0057] Moffett, E. R. , Fryxell, D. C. , Palkovacs, E. P. , Kinnison, M. T. , & Simon, K. S. (2018). Local adaptation reduces the metabolic cost of environmental warming. Ecology, 99, 2318–2326. 10.1002/ecy.2463 30030930

[ece38433-bib-0058] Moran, E. V. , Hartig, F. , & Bell, D. M. (2015). Intraspecific trait variation across scales: implications for understanding global change responses. Global Change Biology, 22(1), 137–150. 10.1111/gcb.13000 26061811

[ece38433-bib-0059] Muñoz, D. J. , Hesed, K. M. , Grant, E. H. C. , & Miller, D. A. W. (2016). Evaluating within‐population variability in behavior and fitness for the climate adaptive potential of a dispersal‐limited species, *Plethodon cinereus* . Ecology and Evolution, 6, 8740–8754. 10.1002/ece3.2573 28035265 PMC5192747

[ece38433-bib-0060] Naya, D. E. , Veloso, C. , & Bozinovic, F. (2008). Physiological flexibility in the Andean lizard *Liolaemus bellii*: Seasonal changes in energy acquisition, storage and expenditure. Journal of Comparative Physiology B, 178, 1007–1015. 10.1007/s00360-008-0292-6 18626649

[ece38433-bib-0061] Novarro, A. J. , Gabor, C. R. , Goff, C. B. , Mezebish, T. D. , Thompson, L. M. , & Grayson, K. L. (2018). Physiological responses to elevated temperature across the geographic range of a terrestrial salamander. Journal of Experimental Biology, 221, jeb178236. 10.1242/jeb.178236 30072387

[ece38433-bib-0062] Ohlberger, J. (2013). Climate warming and ectotherm body size from individual physiology to community ecology. Functional Ecology, 27, 991–1001. 10.1111/1365-2435.12098

[ece38433-bib-0063] Oostra, V. , Saastamoinen, M. , Zwaan, B. J. , & Wheat, C. W. (2018). Strong phenotypic plasticity limits potential for evolutionary responses to climate change. Nature Communications, 9, 10.1038/s41467-018-03384-9 PMC5843647 29520061

[ece38433-bib-0064] Paul, K. I. , Polglase, P. J. , Smethurst, P. J. , O’Connell, A. M. , Carlyle, C. J. , & Khanna, P. K. (2004). Soil temperature under forests: a simple model for predicting soil temperature under a range of forest types. Agricultural & Forest Meteorology, 121, 167–182. 10.1016/j.agrformet.2003.08.030

[ece38433-bib-0065] Peterman, W. E. , Crawford, J. A. , & Hocking, D. J. (2016). Effects of elevation on plethodontid salamander body size. Copeia, 104, 202–208. 10.1643/OT-14-188

[ece38433-bib-0066] Peterman, W. E. , & Semlitsch, R. D. (2013). Fine‐scale habitat associations of a terrestrial salamander: the role of environmental gradients and implications for population dynamics. PLoS One, 8, 1–11. 10.1371/journal.pone.0062184 PMC3646024 23671586

[ece38433-bib-0067] Peterman, W. E. , & Semlitsch, R. D. (2014). Spatial variation in water loss predicts terrestrial salamander distribution and population dynamics. Oecologia, 176, 357–369. 10.1007/s00442-014-3041-4 25154754

[ece38433-bib-0068] Peterson, M. L. , Doak, D. F. , & Morris, W. F. (2019). Incorporating local adaptation into forecasts of species’ distribution and abundance under climate change. Global Change Biology, 25, 775–793. 10.1111/gcb.14562 30597712

[ece38433-bib-0069] Pettersen, A. K. , White, C. R. , & Marshall, D. J. (2015). Why does offspring size affect performance? Integrating metabolic scaling with life‐history theory. Proceedings of the Royal Society B, 282, 20151946. 10.1098/rspb.2015.1946 26559952 PMC4685814

[ece38433-bib-0070] Pettersen, A. K. , White, C. R. , & Marshall, D. J. (2016). Metabolic rate covaries with fitness and the pace of the life history in the field. Proceedings of the Royal Society B, 283, 20160323. 10.1098/rspb.2016.0323 27226476 PMC4892794

[ece38433-bib-0071] R Core Team . (2018). R: A language and environment for statistical computing. R Foundation for Statistical Computing. http://www.R‐project.org/

[ece38433-bib-0072] Riddel, E. A. , & Sears, M. W. (2015). Geographic variation of resistance to water loss within two species of lungless salamanders: implications for activity. Ecosphere, 6, 86. 10.1890/ES14-00360.1

[ece38433-bib-0073] Riddell, E. A. , Odom, J. P. , Damm, J. D. , & Sears, M. W. (2018a). Plasticity reveals hidden resistance to extinction under climate change in the global hotspot of salamander diversity. Science Advances, 4, eaar5471. 10.1126/sciadv.aar5471 30014037 PMC6047487

[ece38433-bib-0074] Riddell, E. A. , Odom, J. P. , Damm, J. D. , & Sears, M. W. (2018b). Supplementary Materials: Plasticity reveals hidden resistance to extinction under climate change in the global hotspot of salamander diversity. Science Advances, 4, eaar5471. 10.1126/sciadv.aar5471 30014037 PMC6047487

[ece38433-bib-0075] Rohr, J. R. , Civitello, D. J. , Cohen, J. M. , Roznik, E. A. , Sinervo, B. , & Dell, A. I. (2018). The complex drivers of thermal acclimation and breadth in ectotherms. Ecology Letters, 21, 1425–1439. 10.1111/ele.13107 30009486

[ece38433-bib-0076] Savage, A. V. M. , Gillooly, J. F. , Brown, J. H. , West, G. B. , Charnov, E. L. , & Savage, V. M. (2004). Effects of body size and temperature on population growth. American Naturalist, 163, 429–441. 10.1086/381872 15026978

[ece38433-bib-0077] Seebacher, F. , White, C. R. , & Franklin, C. E. (2014). Physiological plasticity increases resilience of ectothermic animals to climate change. Nature Climate Change, 5, 61–66. 10.1038/nclimate2457

[ece38433-bib-0078] Sexton, J. P. , McIntyre, P. J. , Angert, A. L. , & Rice, K. J. (2009). Evolution and ecology of species range limits. Annual Review of Ecology Evolution and Systematics, 40, 415–436. 10.1146/annurev.ecolsys.l

[ece38433-bib-0079] Sinclair, B. J. , Marshall, K. E. , Sewell, M. A. , Levesque, D. L. , Willett, C. S. , Slotsbo, S. , Dong, Y. , Harley, C. D. G. , Marshall, D. J. , Helmuth, B. S. , & Huey, R. B. (2016). Can we predict ectotherm responses to climate change using thermal performance curves and body temperatures? Ecology Letters, 19(11), 1372–1385. 10.1111/ele.12686 27667778

[ece38433-bib-0080] Sinervo, B. , Mendez‐De‐La‐Cruz, F. , Miles, D. B. , Heulin, B. , Bastiaans, E. , Villagrán‐Santa Cruz, M. , & Al, E. (2010). Erosion of lizard diversity by climate change and altered thermal niches. Science, 328, 894–899. 10.1126/science.1184695 20466932

[ece38433-bib-0081] Stoks, R. , Geerts, A. N. , & De Meester, L. (2014). Evolutionary and plastic responses of freshwater invertebrates to climate change: realized patterns and future potential. Evolutionary Applications, 7, 42–55. 10.1111/eva.12108 24454547 PMC3894897

[ece38433-bib-0082] Taub, F. B. (1961). The distribution of the red‐backed salamander, *Plethodon c. cinereus*, within the soil. Ecology, 42, 681–698. 10.2307/1933498

[ece38433-bib-0083] Terblanche, J. S. , Nyamukondiwa, C. , & Kleynhans, E. (2010). Thermal variability alters climatic stress resistance and plastic responses in a globally invasive pest, the Mediterranean fruit fly (*Ceratitis capitata*). Entomologia Experimentalis Et Applicata, 137, 304–315. 10.1111/j.1570-7458.2010.01067.x

[ece38433-bib-0084] Urban, M. C. (2015). Accelerating extinction risk from climate change. Science, 348, 571–573. 10.1126/science.aaa4984 25931559

[ece38433-bib-0085] Urban, M. C. , Bocedi, G. , Hendry, A. P. , Mihoub, J.‐B. , Pe’er, G. , Singer, A. , Bridle, J. R. , Crozier, L. G. , De Meester, L. , Godsoe, W. , Gonzalez, A. , Hellmann, J. J. , Holt, R. D. , Huth, A. , Johst, K. , Krug, C. B. , Leadley, P. W. , Palmer, S. C. F. , Pantel, J. H. , … Travis, J. M. J. (2016). Improving the forecast for biodiversity under climate change. Science, 353(6304), aad8466. 10.1126/science.aad8466 27609898

[ece38433-bib-0086] Urban, M. C. , Richardson, J. L. , & Freidenfelds, N. A. (2014). Plasticity and genetic adaptation mediate amphibian and reptile responses to climate change. Evolutionary Applications, 7, 88–103. 10.1111/eva.12114 24454550 PMC3894900

[ece38433-bib-0087] Valladares, F. , Matesanz, S. , Guilhaumon, F. , Araújo, M. B. , Balaguer, L. , Benito‐Garzón, M. , Cornwell, W. , Gianoli, E. , van Kleunen, M. , Naya, D. E. , Nicotra, A. B. , Poorter, H. , & Zavala, M. A. (2014). The effects of phenotypic plasticity and local adaptation on forecasts of species range shifts under climate change. Ecology Letters, 17, 1351–1364. 10.1111/ele.12348 25205436

[ece38433-bib-0088] Wang, L. , Henderson, M. , Liu, B. , Shen, X. , Chen, X. , Lian, L. , & Zhou, D. (2018). Maximum and minimum soil surface temperature trends over China, 1965–2014. Journal of Geophysical Research Atmospheres, 123, 2004–2016. 10.1002/2017JD027283

[ece38433-bib-0089] Welsh, H. H. J. , & Droege, S. (2001). A Case for using Plethodontid salamanders for monitoring biodiversity and ecosystem integrity of North American dorests. Conservation Biology, 15, 558–569.

[ece38433-bib-0090] Winkler, D. W. , Dunn, P. O. , & McCulloch, C. E. (2002). Predicting the effects of climate change on avian life‐history traits. Proceedings of the National Academy of Sciences, 99, 13595–13599. 10.1073/pnas.212251999 PMC129719 12370441

