# Supplementary material for: Geographic variation and thermal plasticity shape salamander metabolic rates under current and future climates
Source: Ecol Evol. 2022 Jan 15;12(1):e8433. doi: 10.1002/ece3.8433 (PMC8809431; doi:10.1002/ece3.8433)
Supplement: Supplementary file 1 — Appendix S1 [file ECE3-12-e8433-s001.docx]

**Appendix S1**


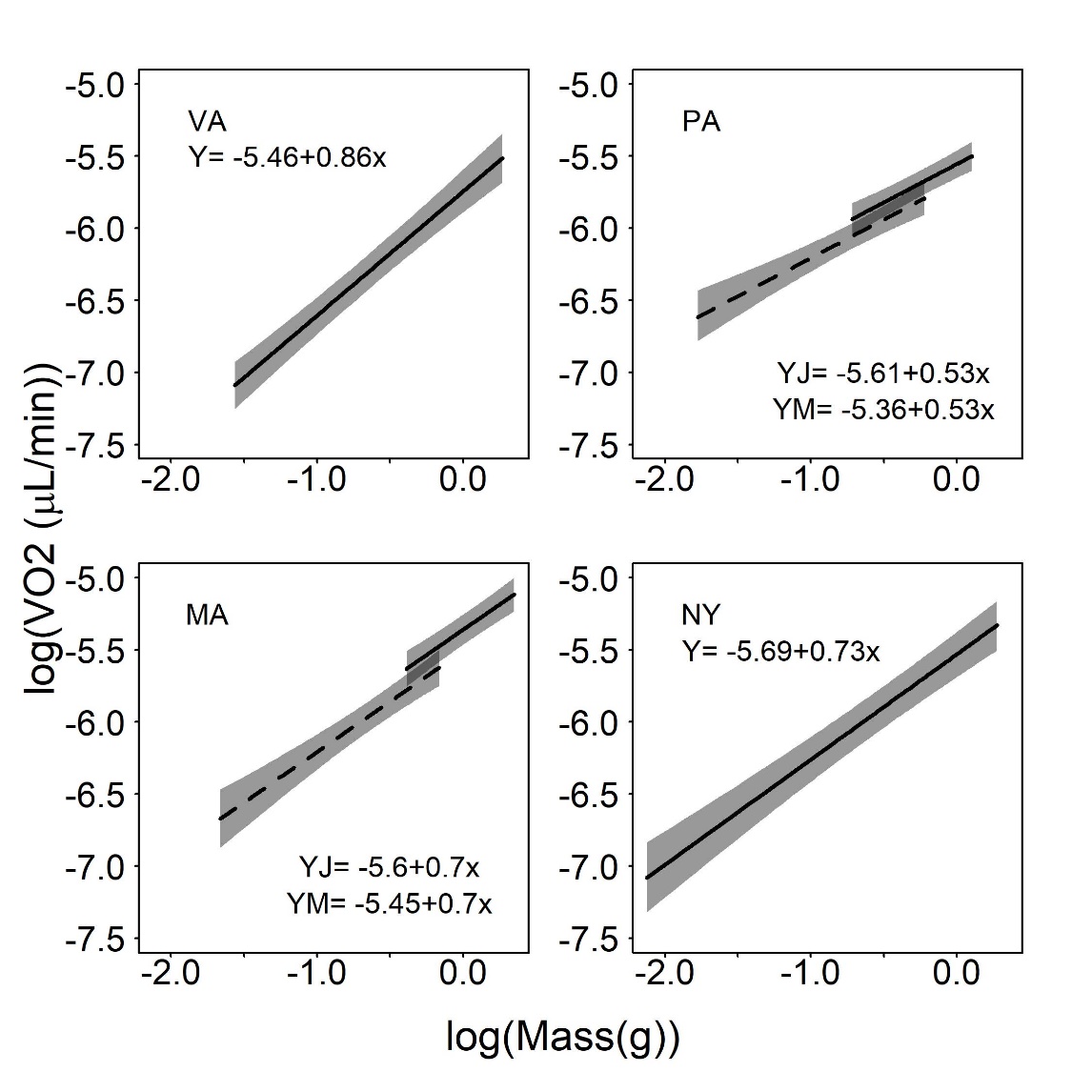
**Body-mass Scaling Results**

Figure S1. Scaling of log(standard metabolic rate) with log(mass). Model selection supported a single scaling relationship in Virginia (VA) and New York (NY). For Pennsylvania (PA) and Massachusetts (MA), scaling varied by life stage (males = solid line, juveniles = dashed line). Scaling equations are given in-frame, and “YJ” is for juveniles and “YM” males.

| Table S1. Mean estimated standard metabolic rate (SMR; µL O_2_/min), with 95% bootstrapped confidence interval, for a typical adult male from each population. Mean SMR vary by thermal regimes (spring, summer, warming). Metabolism was downregulated between spring and summer by Virginia (VA) and Pennsylvania (PA), but not Massachusetts (MA) and New York (NY). Warming and summer confidence intervals overlapped, indicating no clear differences between summer and warming SMR. | | | | | |
| --- | --- | --- | --- | --- | --- |
|  | Spring SMR | Summer SMR | Warming SMR | Spring vs. Summer | Summer vs. Warming |
| VA | 3.17  [2.76, 3.62] | 2.31  [2.04, 2.63] | 2.18  [1.92, 2.5] | -27.2% | -5.7% |
| PA | 3.99  [3.62, 4.37] | 3.16  [2.88, 3.46] | 3  [2.74, 3.3] | -20.6% | -5.0% |
| MA | 3.45  [3.03, 3.92] | 3.61  [3.2, 4.09] | 3.22  [2.87, 3.65] | +4.6% | -10.7% |
| NY | 2.64  [2.29, 3.08] | 3  [2.61, 3.49] | 2.77  [2.4, 3.23] | +13.6% | -7.6% |

**Model Selection Procedures and Results**

*Thermal Plasticity Models* - We performed model selection using maximum likelihood estimates of linear mixed models in R package ‘lme4’. We considered a number of parameters (Table S2), but removed parameters highly collinear with predictors related to our hypotheses. Captivity and cohort had variance inflation factors between 5 and 10, so they were removed. Next, we used Akaike’s Information Criterion (AIC) to determine additive versus interactive relationships between thermal regime and order predictors. Models all included random intercepts and random slopes for individuals during this round of model selection (Table S3). From these 5 models, the best model by AIC had random slopes removed and was compared to the original with random slopes and random intercepts (Table S4). All models performed better without random slopes. The final round of model selection investigated whether life-stage (juvenile vs. male) should be included in the final model (Table S5). This model selection procedure was done independently on each of the four population-specific thermal plasticity models. Once selected, the best final model was computed used restricted maximum likelihood estimators. Final models for each analysis can be found in Table S6. Parameter estimates from each model can be found in Table S7.

*Population Comparison Models* – Under the common thermal regimes (spring and 19.7C), we tested for standard metabolic differences among population, including population-specific allometric relationships (i.e. Population *log(PreMass)). We followed the same selection procedures as described for thermal plasticity models. In Tables S3-S5, replace “TR” with population. In the first round of model selection, we also compared models with and without a population specific allometric relationship (additive and interactive). Otherwise, all procedures were the same.

| Table S2. Name and description of predictors considered for use in linear mixed models. Captivity and Cohort were removed because they were highly correlated with thermal regime (TR). | |
| --- | --- |
| Parameter | Description |
| Log(PreMass) | Log of salamander mass taken before metabolic trial. Continuous. |
| MeanTemp | Mean temperature (C) of the individual salamander’s metabolic trial as measured by a thermocouple. Continuous, z-standardized. |
| TR | The thermal regime the salamander was exposed to prior to metabolic trials. Categorical: spring, summer, warming. |
| Order | The ordinal number representing which day of the metabolic trials a specific trial took place on. Continuous, z-standardized: 1-4. |
| Captivity | The number of days a salamander has been in captivity at the time of metabolic trial. Continuous, z-standardized. |
| Cohort | A categorical variable assigned to each set of salamanders that went through thermal regime assignments together. |
| Individual | A categorical variable identifying each unique salamander. |

| Table S3. List of models considered in first round of model selection using AIC. Random slopes and intercepts are shown using “lme4” notation (random slope\| intercepts and slopes grouped by individual). | | | | | |
| --- | --- | --- | --- | --- | --- |
| Model Name | Model Notation | | | #Fixed Parameters | |
| 1 | Log(PreMass)+MeanTemp+(MeanTemp\|Indv) | | | 2 | |
| 2 | Log(PreMass)+MeanTemp*TR+(MeanTemp\|Indv) | | | 6 | |
| 3 | Log(PreMass)+MeanTemp*TR+Order+(MeanTemp\|Indv) | | | 7 | |
| 4 | Log(PreMass)+MeanTemp+TR +(MeanTemp\|Indv) | | | 3 | |
| 5 | Log(PreMass)+MeanTemp+TR+Order+(MeanTemp\|Indv) | | | 4 | |
| Table S4. The second round of model selection determined whether random intercepts were better than both random slopes and intercepts. Random slopes and intercepts are shown using “lme4” notation (random slope\| intercepts and slopes grouped by individual). The best model from the first round of model selection was used for this comparison. | | | | |  |
| Model Name | | Model Notation | #Fixed Parameters | |  |
| Model (#1-5) | | Log(PreMass)+MeanTemp*TR…+(MeanTemp\|Indv) | *i* | |  |
| Model 6 | | Log(PreMass)+MeanTemp*TR…+(1\|Indv) | *i* | |  |

| Table S5. The third round of model selection determined whether life-stage was an important predictor of plasticity and metabolic rate. Random slopes and intercepts are shown using “lme4” notation (random slope\| intercepts and slopes grouped by individual). The best model from the second round of model selection was used for this round. | | |
| --- | --- | --- |
| Model Name | Model Notation | #Fixed Parameters |
| Model (#1-6) | Log(PreMass)+MeanTemp+TR…+(MeanTemp\|Indv) | *i* |
| Model 7 | Log(PreMass)+MeanTemp*Stage+TR…+(1\|Indv) | *i*+2 |
| Model 8 | Log(PreMass)+MeanTemp+Stage+TR…+(1\|Indv) | *i­+*1 |
| Model 9 | Log(PreMass)+MeanTemp*Stage+TR*Stage…+(1\|Indv) | *i­+*4 |
| Model 10 | Log(PreMass)+MeanTemp+TR*Stage…+(1\|Indv) | *i­+*3 |

| Table S6. Top Models from AIC model selection. All models were >2 AIC away from next model. Conditional coefficients of determination are provided (“R^2^”). There was a total of five models used for final analysis: one for the common thermal regime comparisons, and four for acclimation models. All models explained over half the variation observed. | | |
| --- | --- | --- |
| Analysis | Top Model’s Notation | R^2^ |
| *Common Regime* |  |  |
| 19.7°C | Log(PreMass)+MeanTemp+Pop+Order+(MeanTemp\|Indv) | 0.789 |
| *Acclimation* |  |  |
| Virginia | Log(PreMass)+MeanTemp+TR+Order+(1\|Indv) | 0.547 |
| Pennsylvania | Log(PreMass)+MeanTemp+TR*Stage+Order+(1\|Indv) | 0.796 |
| Massachusetts | Log(PreMass)+MeanTemp*TR+Stage+(1\|Indv) | 0.771 |
| New York | Log(PreMass)+MeanTemp+TR+(1\|Indv) | 0.597 |
|  |  |  |

**Model Parameter Estimates**

| Table S7. Parameter estimates from the linear mixed model analyses are on the natural log scale. The mean estimate, standard error, and bootstrapped 95% confidence intervals are included. The first two models are the common regime analyses (“CR”) followed by the population specific models. Thermal sensitivity is the slope estimate between standard metabolic rate and temperature. Summer and warming regime refer to treatment acclimation regimes. | | | | |
| --- | --- | --- | --- | --- |
|  | Mean Estimate | Std. Error | 2.50% | 97.50% |
| *Spring CR* |  |  |  |  |
| Pennsylvania (intercept) | -5.432 | 0.075 | -5.577 | -5.286 |
| log(PreMass) | 0.766 | 0.079 | 0.612 | 0.920 |
| Thermal Sensitivity | 0.494 | 0.021 | 0.453 | 0.535 |
| Virginia Effect | -0.072 | 0.065 | -0.199 | 0.054 |
| Massachusetts Effect | -0.130 | 0.058 | -0.242 | -0.018 |
| New York Effect | -0.295 | 0.055 | -0.403 | -0.188 |
| Male life-stage Effect | 0.095 | 0.066 | -0.034 | 0.224 |
| Trial Order Effect | 0.059 | 0.021 | 0.017 | 0.100 |
|  |  |  |  |  |
| *19.7 CR* |  |  |  |  |
| Pennsylvania (intercept) | -5.564 | 0.043 | -5.647 | -5.480 |
| log(PreMass) | 0.795 | 0.049 | 0.699 | 0.891 |
| Thermal Sensitivity | 0.468 | 0.022 | 0.423 | 0.511 |
| Virginia Effect | -0.260 | 0.055 | -0.367 | -0.154 |
| Massachusetts Effect | 0.012 | 0.062 | -0.108 | 0.131 |
| New York Effect | -0.117 | 0.061 | -0.236 | 0.002 |
| Male life-stage Effect | 0.083 | 0.021 | 0.042 | 0.124 |
|  |  |  |  |  |
| *Virginia* |  |  |  |  |
| Spring SMR (Intercept) | -5.462 | 0.075 | -5.607 | -5.317 |
| log(PreMass) | 0.858 | 0.064 | 0.734 | 0.983 |
| Thermal Sensitivity | 0.402 | 0.037 | 0.330 | 0.475 |
| Summer Effect | -0.317 | 0.084 | -0.480 | -0.154 |
| Warming Effect | -0.376 | 0.084 | -0.540 | -0.211 |
| Trial Order Effect | 0.081 | 0.037 | 0.009 | 0.152 |
|  |  |  |  |  |
| *Pennsylvania* |  |  |  |  |
| Spring SMR (Intercept) | -5.609 | 0.059 | -5.724 | -5.495 |
| log(PreMass) | 0.531 | 0.070 | 0.392 | 0.667 |
| Thermal Sensitivity | 0.489 | 0.014 | 0.462 | 0.516 |
| Summer Effect | -0.104 | 0.048 | -0.198 | -0.009 |
| Warming Effect | -0.132 | 0.048 | -0.225 | -0.038 |
| Male life-stage Effect | 0.246 | 0.060 | 0.130 | 0.362 |
| Trial Order | 0.076 | 0.014 | 0.050 | 0.103 |
| Summer-Male Interaction Effect | -0.128 | 0.068 | -0.261 | 0.006 |
| Warming-Male Interaction effect | -0.149 | 0.067 | -0.281 | -0.018 |
|  |  |  |  |  |
| *Massachusetts* |  |  |  |  |
| Spring SMR (Intercept) | -5.598 | 0.058 | -5.710 | -5.486 |
| log(PreMass) | 0.699 | 0.082 | 0.541 | 0.857 |
| Thermal Sensitivity | 0.553 | 0.028 | 0.498 | 0.608 |
| Summer Effect | 0.052 | 0.040 | -0.026 | 0.129 |
| Warming Effect | -0.062 | 0.040 | -0.139 | 0.016 |
| Male life-stage Effect | 0.148 | 0.064 | 0.023 | 0.272 |
| Summer-Thermal Sensitivity Interaction | -0.082 | 0.039 | -0.159 | -0.005 |
| Warming-Thermal Sensitivity Interaction | -0.075 | 0.040 | -0.153 | 0.002 |
|  |  |  |  |  |
| *New York* |  |  |  |  |
| Spring SMR (Intercept) | -5.695 | 0.052 | -5.796 | -5.594 |
| log(PreMass) | 0.729 | 0.061 | 0.610 | 0.849 |
| Thermal Sensitivity | 0.458 | 0.023 | 0.414 | 0.502 |
| Summer Effect | 0.127 | 0.057 | 0.016 | 0.239 |
| Warming Effect | 0.048 | 0.057 | -0.063 | 0.160 |
